# Transmission of MRSA between Companion Animals and Infected Human Patients Presenting to Outpatient Medical Care Facilities

**DOI:** 10.1371/journal.pone.0026978

**Published:** 2011-11-10

**Authors:** Jorge Pinto Ferreira, Kevin L. Anderson, Maria T. Correa, Roberta Lyman, Felicia Ruffin, L. Barth Reller, Vance G. Fowler

**Affiliations:** 1 Department of Population Health and Pathobiology (PHP), North Carolina State University (NCSU) College of Veterinary Medicine, Raleigh, North Carolina, United States of America; 2 Department of Infectious Diseases, Duke University School of Medicine, Durham, North Carolina, United States of America; University of Iowa, United States of America

## Abstract

Methicillin-resistant *Staphylococcus aureus* (MRSA) is a significant pathogen in both human and veterinary medicine. The importance of companion animals as reservoirs of human infections is currently unknown. The companion animals of 49 MRSA-infected outpatients (cases) were screened for MRSA carriage, and their bacterial isolates were compared with those of the infected patients using Pulsed-Field Gel Electrophoresis (PFGE). Rates of MRSA among the companion animals of MRSA-infected patients were compared to rates of MRSA among companion animals of pet guardians attending a “veterinary wellness clinic” (controls). MRSA was isolated from at least one companion animal in 4/49 (8.2%) households of MRSA-infected outpatients vs. none of the pets of the 50 uninfected human controls. Using PFGE, patient-pets MRSA isolates were identical for three pairs and discordant for one pair (suggested MRSA inter-specie transmission p-value = 0.1175). These results suggest that companion animals of MRSA-infected patients can be culture-positive for MRSA, representing a potential source of infection or re-infection for humans. Further studies are required to better understand the epidemiology of MRSA human-animal inter-specie transmission.

## Introduction

The epidemiology of methicillin-resistant *Staphylococcus aureus* (MRSA) is dynamic [1], [2]. First identified in the 1960s, MRSA was initially considered a nosocomial pathogen. Beginning in the late 20^th^ century, a specific clone of MRSA, known as USA300, emerged as a leading cause of community-acquired infection [3]–[5]. Recently, another strain of MRSA, Sequence Type 398 (ST-398), has been shown to be strongly associated with livestock [6], accounting for up to 20% of all human cases of MRSA infection in the Netherlands [7].

During this time, a growing number of reports have described probable transmission of *S. aureus* and MRSA, in particular, between humans and companion animals [8]–[13]. Little is known, however, about the potential role of companion animals in the transmission of MRSA to humans. For example our understanding regarding direction of transmission, persistence of colonization, rate of animal-human transmission, inter-specie transmission risk factors, animal population or breeds with increased risk to be carriers of MRSA and the significance of companion animals as reservoirs for human MRSA infections are all incomplete.

In the current study, we sought to investigate the significance of pets/companion animals as sources of MRSA infection or re-infection for human outpatients by evaluating MRSA transmission between MRSA-infected outpatients and their companion animals. Our results suggest that this reservoir might be more significant than currently considered.

## Materials and Methods

### Ethics Statement

This cross-sectional study was a collaboration between Duke University School of Medicine and North Carolina State University College of Veterinary Medicine and was approved by Institutional Review Boards (CR1_Pro00018484; 1417-10) and Animal Care and Use Committees ( A-329-09-11; 10-054-B) at both participating institutions.

### Ascertainment of Cases and Control Groups

Between January and May 2010, MRSA-positive patients seen as outpatients at a large southeastern United States hospital were identified. Other inclusion criteria were an age of 18 years or older, ability to speak in English and residence within a 50 miles radius from the hospital. The health care providers of the patients meeting these criteria were contacted by study personnel to obtain permission to contact the individuals. If the health care provider consented, patients were contacted by phone to determine if they had companion animals. If patients lived with companion animals and consented (in written form) to participate in the study, a household visit was scheduled to obtain nasal swabs from the animals to determine their MRSA status. A short questionnaire was given to the animal guardians on the day of the visit. The goal of this questionnaire was to identify inter-specie transmission risk factors. Forty nine patients, 76 dogs, 25 cats and3 hamsters were included in the study population. Thirteen adult (older than eighteen) family members (of the 49 human cases) voluntarily participated in this study, answering the questionnaire and self-collecting nasal swabs to determine their MRSA status.

Companion animals presenting to a veterinary institution wellness clinic and their guardians served as a control population. Animals were voluntarily taken to this clinic mainly for prophylactic vaccinations, being otherwise generally healthy. The control population included 50 people and 45 dogs and 30 cats.

We used contingency tables to assess the associations between case/control status and the exposure/demographic variables. Counts, percentages and odds ratios were calculated to quantitate the strengths of these associations and the statistical significance was determined with Fisher's exact test. Statistical analysis was performed with SAS 9.2 (SAS Institute, Cary, NC, USA).

### Microbiological identification of MRSA isolates

The clinical human MRSA isolates from the patients were collected from the Clinical Microbiology Laboratory of the medical school integrated in this project and stored (−80°C) until required for additional use.

*Staphylococcus* spp. identification was performed in accordance with routine laboratory techniques, including typical colony morphology, gram stain, catalase and coagulase tests. *S. aureus* and *S. pseudintermedius* diagnosis was confirmed by multiplex PCR [14]. Resistance to oxacillin and cefoxitin was determined using standard disk diffusion [15]. *S. aureus* isolates were classified as MRSA if the inhibition zone was less than or equal to 21 mm for cefoxitin or less than or equal to 10 mm for oxacillin [15]. Oxacillin was used to determine susceptibility of the *S. pseudintermedius* isolates. When the inhibition zone was less than or equal to 17 mm, they were considered resistant.

*mec*A PCR was performed on the human and animal MRSA isolates [16].

Genetic relatedness was evaluated by use of pulsed field gel electrophoresis (PFGE) and *spa* typing, as previously described [17], [18].

## Results

A total of 49 MRSA-infected outpatients (cases) and 50 uninfected (human) controls participated in the study. The animal case population was larger than the control population (total of 107 vs 75 animals) and included more dogs than the animal control population (76 vs. 45).

Four out of the 49 (8.2%) human cases with culture-confirmed MRSA infections lived with a companion animal (2 dogs, 1 cat, 1 hamster) from which MRSA was isolated. One of the patients diagnosed with MRSA lived with a methicillin-resistant *Staphylococcus pseudintermedius* (MRSP) positive dog.

No MRSA or MRSP was found in the 13 family members of the MRSA-infected patients that voluntarily participated in this study, or in the 50 humans or 75 animals of the control population.

**Figure 1:**
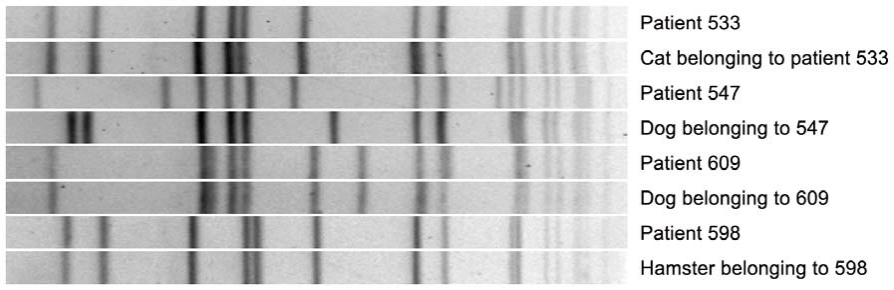
PFGE comparison of human and animal MRSA pairs.

Using PFGE, three of the human-animal MRSA pairs were identical and one was discordant (figure 1). Three of the four human-animal MRSA isolates pairs were classified as *spa* type 2 and clonal complex 5 (table 1).

**Table 1:** Summary of the classification of the MRSA isolates, using *spa* typing.

patient : animal pair	CDC classification	*spa* typing	clonal complex	Pair similarity	Specific risk factor(s)
patient 533cat 533	USA 100USA 100	type 2type 2	cc 5cc 5	identical	patient was cancer survivor and had been hospitalized in the previous year; animal was allowed to move freely in house
patient 547dog 547	USA 300not a common CDC-designated isolate	type 1type176	cc 8cc 5	Non identical	patient had been hospitalized in the previous year and animal was allowed to move freely in the house
patient 598hamster 598	not a commonCDC-designated isolatenot a commonCDC-designated isolate	type 2type 2	cc 5cc 5	identical	patient with diabetes, organ transplant, renal insufficiency and depression that had been hospitalized in the previous year; animal with open sores
patient 609dog 609	not a commonCDC-designated isolatenot a commonCDC-designated isolate	type 2type 2	cc 5cc 5	identical	patient was a healthcare worker and animal was allowed to move freely in the house

Table 2 presents the results of the univariable analysis (based on the questionnaire answers) of the variables potentially associated with MRSA carriage and human-animal transmission. The ones that were significantly different between cases and controls are highlighted.

**Table 2:** Univariable analysis (based on the questionnaire answers) of the variables potentially associated with MRSA carriage and human-animal transmission.

Variable	Cases(n ; %)	Controls(n ; %)	OR	95% CI
Do you have a FM who is HCW?				
*Yes*	7 (14.28%)	17 (34%)		
*No*	42 (85.71%)	33 (66%)	0.32	[0.12, 0.87]
Do you have a FM who is a veterinarian?				
*Yes*	1 (2.27%)	9 (18%)		
*No*	43 (97.72%)	41 (82%)	0.11	[0.01, 0.87]
**Are there children in the household?**				
***Yes***	**22 (44.9%)**	**8 (16%)**		
***No***	**27 (55.1%)**	**42 (84%)**	**4.28**	**[1.67, 10.98]**
Has a FM been treated with AB in the past year?				
*Yes*	22 (44.9%)	14 (29.79%)		
*No*	27 (55.1%)	33 (70.21%)	1.92	[0.83, 4.45]
**Has a FM been diagnosed with MRSA in the past year?**				
***Yes***	**8 (16.33%)**	**1(2.04%)**		
***No***	**41 (83.67%)**	**48 (97.96%)**	**9.37**	**[1.12, 78.05]**
**Were you hospitalized in the past year?**				
***Yes***	**15 (31.25%)**	**4 (8%)**		
***No***	**33 (68.75%)**	**46 (92%)**	**5.23**	**[1.59, 17.18]**
**Have you been diagnosed with a disease or take medication that affects your immune condition?**				
***Yes***	**28 (57.14%)**	**3 (6%)**		
***No***	**21 (42.86%)**	**47 (94%)**	**20.89**	**[5.71, 76.42]**
Are you a HCW?				
*Yes*	8 (16.33%)	3 (6%)		
*No*	41 (83.67%)	47 (94%)	3.06	[0.76, 12.29]
Aware of recent (past month) contact with person or animals MRSA positive?				
*Yes*	7 (14.29%)	5 (10%)		
*No*	42 (85.71%)	45 (90%)	1.5	[0.44, 5.09]
**Were you treated with any AB in the past year?**				
***Yes***	**38 (77.55%)**	**18 (36%)**		
***No***	**11 (22.45%)**	**32 (64%)**	**6.14**	**[2.53, 14.89]**
Do any of your animals have current sores?				
*Yes*	7 (14.28%)	6 (12%)		
*No*	42 (85.71%)	44 (88%)	1.22	[0.34, 3.51]
Were any of your animals hospitalized in the past year?				
*Yes*	5 (10.20%)	6 (12%)		
*No*	44 (89.80%)	44 (88%)	0.83	[0.26, 3.25]
Are any of your animals allowed to go outdoors?				
*Yes*	24 (48.98%)	11 (22%)		
*No*	25 (51.02%)	39 (78%)	3.4	[0.71, 4.07]
Are any of your animals allowed to move freely in the house?				
*Yes*	36 (74%)	46 (92%)		
*No*	13 (26%)	4 (8%)	0.24	[0.16, 1.79]
Are any of the animals allowed to lick human faces?				
*Yes*	21 (42.86%)	37 (74%)		
*No*	28 (57.14%)	13 (26%)	0.26	[0.24, 1.31]
Are any of the animals allowed to sleep where humans sleep?				
*Yes*	31 (63.27%)	37 (74%)		
*No*	18 (36.73%)	13 (26%)	0.61	[0.34, 1.90]
Do you have contact with your animals everyday?				
*Yes*	42 (85.71%)	45 (88.89%)		
*No*	7 (14.29%)	5 (11.11%)	1.5	[0.35, 4.05]

The ones that were significantly different between cases and controls are highlighted. “Don't know” or “missing” answers were excluded from the analysis. Legend: FM = family member; HCW = health care worker; AB = antibiotic.

## Discussion

Our results provide further evidence into the potential significance of companion animals as a source of infection and/or re-infection of humans/outpatients. These findings are particularly important, as MRSA is the most common identifiable cause of soft tissue infection in the US [3] and it is estimated that about 75 million dogs and 88 million cats are owned in the US [19]. Because companion animals are increasingly seen and treated as family members by their guardians [20], the opportunity for transmission between humans and pets is only likely to increase. Our results are consistent with previous reports. Weese *et al.* (2006) studied the transmission of MRSA in veterinary clinics and in the households, after the identification of a MRSA positive animal. These authors described 6 cases. MRSA was isolated from 16% (14/88) of household contacts or veterinary personnel and in all of the 6 cases it was possible to find at least one human isolate identical to the animal (initial) one [21]. More recently, Faires *et al.* evaluated both the rate of MRSA transmission from infected animals to humans and vice-versa. When the MRSA-infected animal was initially identified, at least one MRSA-colonized person was identified in over one-quarter (6/22; 27.3%) of the study households. By contrast, only one of the 8 (12.5%) study households of MRSA-infected humans contained a MRSA-colonized pet [22]. By evaluating about 5 times the number of MRSA-infected humans as Faires *et al.* and finding a similar companion animal MRSA colonization rate (∼8%), the current study externally validates the findings of the previous study. Our results clearly demonstrate that MRSA transmission between infected patients and companion animals occurs. Such transmission between humans and animals has been previously implicated as potential cause of recurrent MRSA infections [8]–[13]. Previous publications have described cases where human MRSA could not be linked with traditional MRSA sources in the community or health care facilities [23]. This challenges the accepted epidemiology of MRSA and suggests that there are currently unrecognized/unknown sources of MRSA. Finding 5 out of 8 (62.5%) MRSA isolates that were not identical to any of the most common (and previously described by the Centers for Disease Control (CDC)) Hospital Acquired (HA) or Community Acquired (CA) MRSA clones seems to reinforce this idea.

Not finding MRSA in any of the humans or animals of the control population was surprising. Veterinarians have been described as a professional group with increased risk of carrying MRSA [7], [24]. Different prevalence studies have found very diverse prevalence values in small/companion animals [25]–[28]. To our knowledge, prevalence in companion animals has never been determined in North Carolina, which makes it hard to evaluate the absence of MRSA in the animal control population.

Our study has limitations. Finding MRSA in both outpatients and their companion animals is suggestive of inter species transmission of this agent. However, we can only speculate about transmission and there is the possibility that both parts became infected from different sources. Direction of transmission also cannot be determined. Finding 3 concordant human-animal MRSA pairs is not statistically significant (p = 0.1175) considering a reasonable significance level and therefore a larger sample size should be considered in future studies. The most ideal control population would have been the one formed by outpatients diagnosed with methicillin sensitive *Staphylococcus aureus* (MSSA) living with companion animals, with the same number of both humans and animals in the study and control populations (a 1∶1 ratio). Using the population of animals and their guardians that attended a wellness clinic was, therefore, a convenient, involving less costs and more readily available choice. We still believe, however, that this gave us an estimate of the prevalence of MRSA co-existence at the household level in healthy humans and animals in the general population. The average time between a MRSA outpatient identification (control) and sampling/swabbing of its companion animals was approximately one month, so there is a possibility that some colonized animals were missed [22].

### Other Staphylococcus spp. trans-infection

The primary goal of this project was to study human-animal MRSA transmission. Increased attention has, at the same time, been given by the scientific community to other *Staphylococcus* species (spp.) inter-specie transmission [29]–[32]. More recently, a novel staphylococcus has been identified: *Staphylococcus pseudintermedius* [33]. Since *S. pseudintermedius* is coagulase positive, the possibility of misdiagnosis in clinical microbiology laboratories is possible and has to be taken into consideration [31], [34]. Our finding of a human infected with MRSA living with an MRSP animal should be investigated in future projects. The exchange of genetic material between different species of staphylococci has been repeatedly reported and emphasized [32], [35], [36] and its significance for human infections is currently unknown.

### Challenges and future research

One of the most challenging aspects of this project was the enrollment of patients. Of the 557 patients diagnosed with MRSA during our study at the medical school hospital integrated in this project, 231 would match our inclusion criteria and only 49 were enrolled (response rate of approximately 21% (49/231)).Reasons for this included: difficulty in reaching the health care providers and patients, the non-existence of companion animals in the household, residences being outside the 50 mile radius, the inexistence of financial compensation to the participants, and patient or medical team declining participation.

Future research should focus on the dynamics of transmission. Longitudinal studies with multiple samplings of animals and humans will be critical in addressing questions regarding direction of transmission and duration of colonization. Obtaining an IRB permission for the enrollment and sampling of children would be important, as MRSA is known to be more prevalent in younger kids [37]. Environmental samples should also be taken at the household level to identify other potential sources of reinfection. Staphylococcus diagnostic protocols should be carefully reviewed to make sure that the recently discovered coagulase positive staphylococci are included in the differential diagnosis list. Staphylococci should be characterized at the molecular level with different techniques (PFGE, multiplex PCR, multi locus sequence typing, *spa* typing) to allow a better comparison with different studies and traceability of the isolates origin.

### Conclusions

Nearly 8% of MRSA outpatients lived with a MRSA pet. When faced with chronic and or recurrent MRSA cases, physicians should consider the possibility of household pets as MRSA source. Patients should be informed of this possibility. Unnecessary close contact should be avoided and heightened hygiene practices should be instituted. Sampling/swabbing of all the human and animals in a household seems appropriate to identify unrecognized sources and break potential cycles of reinfection especially in cases involving immunocompromised patients. It is critical that medical and veterinary institutions partner and collaborate in researching this topic. The legal/institutional approval that regulates this type of partnerships should be expedited to encourage them. MRSA epidemiology is a perfect example of an infectious disease agent whose control requires a “One Health” approach.
